# Editorial: The Role of Inhibitory Receptors in Inflammation and Cancer

**DOI:** 10.3389/fimmu.2020.633686

**Published:** 2020-12-22

**Authors:** Ali A. Zarrin, Renato C. Monteiro

**Affiliations:** ^1^TRexBio, South San Francisco, CA, United States; ^2^Center for Research on Inflammation, Université de Paris, Paris, France

**Keywords:** inhibitory receptor, cancer, inflammatory disease, ITIM, immunoreceptor tyrosine-based inhibitory motif, ITAM

Inhibitory signals play an essential role in the control of immune responses allowing to preserve self while eliminating the insults. They are crucial to prevent tissue injury induced by overactivation of the immune system and to maintain homeostasis (1). They are delivered by receptors primarily expressed on lymphoid and myeloid cells that allow the pairing of activation and inhibition necessary to initiate, amplify, and then terminate immune responses. The importance of both type of receptors has been illustrated for example by paired Ig-like type receptors (PILR) in which inhibitory receptor and its counterpart activating receptor are coexpressed on single myeloid cells (2). Impairment of these signals increases the chance of developing chronic inflammation and autoimmunity and in contrast, tools activating such receptors could be beneficial to reduce inflammation (3). Genetic variants of certain immunoreceptors (IRs) validate their important role in the development of human disease (4).

The human genome contains more than 300 genes encoding potential inhibitory receptors, of which more than 60 have been functionally characterized over the past decades (1). Negative signaling in the immune system classically involves phosphatases and anti-inflammatory cytokines which prevent or abort kinase-dependent activating signaling (5). Inhibitory receptors containing immunoreceptor tyrosine-based inhibition motif (ITIM) in their cytoplasmic domain co-aggregate with immunoreceptor tyrosine-based activation motif (ITAM)-containing activating receptors during activation (5). Kinases associated with ITAMs induce the tyrosine phosphorylation of juxtaposed ITIMs, resulting in phosphatase recruitment to the phosphorylated ITIMs to abrogate the ITAM-dependent positive signal (6). ITAM motifs are present in essential receptors of the immune system such as T- and B-cell antigen receptors (TCR, BCR) and Fc receptors (FcR) as well as in an expanding family of ITAM-associated receptors with various functions in both innate and adaptative immunity. The main phosphatases implicated in inhibitory receptor signals are tyrosine phosphatases (e.g., SHP-1) or phosphatidylinositol phosphatases (e.g., SHIP-1). Once recruited they are ideally localized to find their respective substrates (e.g., kinases, adaptor proteins, signal effector molecules, or their membrane lipid anchors) to impede ITAM-initiated signaling.

Besides ITIM, ITAM motif can also propagate inhibitory signaling to heterologous activating receptors, being named, in this configuration, inhibitory ITAM (ITAMi) (6, 7). Some FcR (CD16A, CD32A, CD89) or TREM2, are associated with the ITAM-bearing adaptors FcRγ or DAP12 and act as bi-functional receptors that can trigger inhibitory signals toward a whole array of activating receptors (7). The ITAMi signals are initiated by targeting ITAM-containing FcR at low valency and is operative at distance independent of a co-aggregation mechanism, hence without requirement of signals initiated by the activating receptors. They recruit the tyrosine phosphatase SHP-1. Such dual receptor functions are controlled by Src kinases (8). They have been observed for other ITAM-bearing receptors including several innate immune receptors, suggesting that this could represent a widespread mechanism of immune regulation. Thus, a selective pressure during evolution may have generated single switch molecules capable of mediating either activation or inhibition depending on the type/valency of the ligand.

Yet, despite the clear protective role of inhibitory receptors, dysregulated inhibitory signals can be deleterious to the host as shown by the blockade of T lymphocyte responses to tumors. For example, tumor-specific T cells that exhibit an exhausted, unresponsive phenotype express high levels of inhibitory receptors such as CTLA4, PD1, and LAG3 (9, 10), and intra-tumoral regulatory T cells promote immunosuppression through expression of multiple inhibitory receptors. Overcoming this inhibitory receptor-mediated immune tolerance thus has been a major focus of recent cancer immunotherapeutic developments promoting enormous progress for patient treatment that have been rewarded by the Nobel prize in Physiology or Medicine in 2018.

Inhibitory signals are also exploited by microorganisms. For example, bacteria and parasites can evade the immune system by eliciting ITAMi signal through FcR (CD16) or C-type lectins (Mincle) on phagocytes leading to uncontrolled systemic infection, sepsis or parasite invasion (11, 12). In the case of CD16A, its direct targeting by *E. coli* on macrophages strongly inhibited phagocytosis through scavenger receptor MARCO. The ITAMi signal involved SHP-1 recruitment to CD16A-FcRγ and resulted in the reduced phosphorylation of phosphoinositide 3-kinase (PI3K), a key signal effector molecule in phagocytosis.

In this volume, Crute et al. present a new method for identification of inhibitory receptors that bind to specific SH2 domains, termed Inhibitory Receptor Trap (IRT). The authors use immunoblotting to show that IRT works to selectively isolate SH2 interacting partners and ultimately providing mass spectrometry as a read-out to identify unexpected interacting partners (Crute et al.). As an example, they have identified novel candidates following interaction between SHIP and PD-1. This new method should find broad use in many cell biology investigations.

Sivori et al. contribute with an overview on NK cell function that is finely regulated by HLA-specific inhibitory receptors such as killer Ig-like receptors (KIR) and non-HLA inhibitory receptors such as Siglecs which discriminate between HLA and non-HLA antigens in healthy cells and tumor or virus-infected cells. They discussed how ITIM-bearing receptors such as Siglec 7, LAIR-1, and IRp60, recognize ligands including sialic acids, extracellular matrix/collagen, or aminophospholipids. These ligands can be expressed at the surface of tumor cells, thus inhibiting NK cell function, which can further be blocked by expression of the PD-1 on NK cells induced by cytokines with cortisol, a combination which may occur in the microenvironment of various tumors (Sivori et al.). The authors provide new perspectives on how to block inhibitory receptor checkpoints on NK cells to restore their anti-tumor activity for tumor immunotherapy. The mechanisms responsible for immune checkpoint inhibitor resistance remain incompletely understood. Liu et al. utilize a computational approach to show that the immune inhibitors and immune stimulators were positively and concomitantly correlated with PD-1 expression. Authors suggest that there might be a regulatory interaction among these immune receptor hubs which may promote relapses.

Davis provides an example of such a scenario in the context of human cancer where expression of certain IRs are associated with PD1 and highlights the utility of Fc receptor-like (FCRL1–6) gene family as a therapeutic and/or biomarker target. FCRL1–6 genes encode type I transmembrane glycoproteins with cytoplasmic ITAM or ITIM motifs (Davis). FCRL1–5 are preferentially expressed by B cells and modulate B cell antigen-receptor-mediated signaling (Davis). In contrast, FCRL6 is expressed by mature T cell and NK subpopulations with cytotoxic potential (Davis). Its restricted expression and extracellular interactions with MHCII/HLA-DR, is emerging as an important regulatory axis in tolerance and cancer immunity (13). FCRL6 is upregulated in HLA-DR+ tumor samples from melanoma, breast, and lung cancer patients who relapsed following PD-1 blockade (13). Thus, FCRL6 may serve as a unique inhibitory receptor to counteract the productive immunity in cancer.

Leukocyte-associated immunoglobulin-like receptor 1 (LAIR-1) is an immune inhibitory receptor which *in vitro* binds to collagen and collagen domain containing proteins including surfactant protein D and C1q as well as epithelial cellular adhesion molecule (Ep-CAM) (1). LAIR-1 recruits SHP-1 and SHP-2 phosphatases upon activation, and cross-linking of the LAIR-1 on NK cells or T cells results in strong inhibition of NK or T cell–mediated cytotoxicity (1). Carvalheiro et al. report that LAIR1 is expressed in skin CD14+ cells, macrophages and CD1c+ DCs. Authors show that LAIR1 ligation with anti-LAIR1 antibody in monocytes, inhibits toll-like receptor (TLR)4- and Interferon (IFN)-α-induced signals suggesting that LAIR1 could act as a negative regulator of inflammatory response under certain pathogenic cues (Carvalheiro et al.). The authors provide comprehensive kinetics and gene expression studies on how LAIR1 is regulated under different stimulatory conditions in monocyte-derived macrophages and monocyte-derived DCs (Carvalheiro et al.). LAIR1 is upregulated in wound healing preclinical models and its expression is correlated with increased macrophage markers (Carvalheiro et al.). Interestingly, soluble LAIR-1 and/or LAIR-2 are increased in inflammatory diseases such as rheumatoid arthritis, Graves’ disease and autoimmune thyroiditis (14, 15). In preclinical cancer models, LAIR1-collagen interactions promote CD8 T cell exhaustion and the combination of PD-1 blockade with LAIR2 over-expression reduces lung tumor growth and metastasis (16). Thus, LAIR1 may have an important function in different immune cells against broad inflammatory and cytotoxic pathways to control productive immunity.

Signal regulatory protein alpha (SIRPα) is another inhibitory immunoreceptor expressed on myeloid and neuronal cells. SIRPα interacts with CD47 which is expressed broadly in different tissues (17). Upon phosphorylation the SIRPα ITIM acts to recruit and activate the tyrosine phosphatases SHP-1 and/or SHP-2, which inhibit tyrosine phosphorylation-dependent signaling events and the resulting downstream cellular effector functions, including, e.g., phagocytosis (17). CD47-SIRPα axis forms an important innate immune checkpoint, with CD47 acting as so-called “don’t-eat-me” signal, which prevents the engulfment of healthy cells by myeloid cells. Cancer cells may highjack this pathway by over-expressing CD47 thus escaping immune-mediated destruction (17). Franke et al. show that SIRPα on B1 cells negatively regulates their migration, B1 cell numbers in the spleen, and systemic natural antibody production, without directly affecting B1 cell activation. B1 cells are a subset of B cells that is the main source of natural low affinity antibodies. Authors utilize the mice lacking the cytoplasmic tail of SIRPα (SIRPαΔCYT mice) in their hematopoietic compartment and show that they are protected against atherosclerosis with increased natural antibody levels against oxidized lipids (Franke et al.). Additional studies with SIRPα B cell specific conditional knockout might help to better dissect the function of this inhibitory receptor in B cells trafficking. Thus, novel functions may emerge depending on cellular context for various IRs adding another level of complexity to understand their biology.

Alfarra et al. provide in-depth on clinical applications of IRs for multiple myeloma (MM) focusing on Natural killer (NK) cell. NK cells are an intriguing immune cell type in MM given promising results of elotuzumab (anti-SLAMF7) and daratumumab (anti-CD38) that enhance NK cell-mediated anti-tumor cell toxicity by activating the antibody-dependent cellular cytotoxicity (ADCC) mechanism (Alfarra et al.). Although these mAbs have improved the clinical outcomes of both newly diagnosed and relapsed or refractory MM (RRMM) patients, only a subgroup of patients responds to these mAbs, highlighting the complexity of the disease. CAR-NK cell therapies and combinations of existing treatments also work to restore the innate killing capacity of NK cells in MM. Authors provide a detailed review and their perspectives summarizing the advances made in this area and highlight the utility of several NK cell IRs including several molecules such as CD38, CD138, SLAMF7, SLAMF3, CD56, NKG2D, and BCMA (Alfarra et al.).

Immunoglobulin G4-related disease (IgG4-RD) is a systemic multiorgan autoimmune condition of unknown cause characterized by enhanced serum IgG4 antibodies and tissue expressing plasma cells accompanied by immunopathology associated with altered acquired immunity (18). During active IgG4-RD, the expansion of circulating plasmablasts, T cells, and eosinophils plus autoantibodies are detected. Recent transcriptome studies have revealed some insights into the molecules and pathways that might be involved in IgG4-RD (18). Several cytokines such as Th2-related, T follicular helper cell (Tfh)-related and Treg-related transcripts have been reported (18). In this issue, Cai et al. utilized an unbiased proteomic approach plus existing transcriptome data to study how different pathways/genes might be altered in IgG4-RD comparing both serum and tissue samples from naive IgG4-RD patients and healthy volunteers. The authors identify multiple signaling pathways that are dysregulated including MAPK, PI3K-Akt, Ras, TGF-β, NF-κB, and Rap1 modules (Cai et al.). They also report modulation of Fc gamma receptor (FcγR) and increased IgG3 levels which may potentiate phagocytosis, antigen presentation, as well as FcγR-mediated systemic pathology through inhibitory and activating receptors (5). This analysis plus availability of tool molecules against candidate pathways might help to model pathogenic pathways *in vitro* and ultimately to identify druggable targets that could be beneficial in IgG4-RD patients.

Complex interactions between immune inhibitors and immune stimulators in various cell types regulate productive immunity in inflammation and cancer. Given the success of checkpoint immunotherapies, we are beginning to better understand the function and dominant effect of each inhibitory receptor in humans. It has been more and more appreciated that understanding disease heterogeneity and underlying immune pathology is critical to enable effective therapeutics. Profiling tumor microenvironment or inflammatory disease tissues using high resolution gene expression, multi-omics, and multiparameter histological studies combined with functional studies should help to point to rationale therapeutic approaches in different disease areas. Functional characterization of various inhibitory receptors in different immune cell subsets should reveal novel therapeutics or essential biomarkers to track these important regulatory molecules in the immune system.

## Author Contributions

Both authors contributed equally to finalize the manuscript. All authors contributed to the article and approved the submitted version.

## Funding

RCM laboratory is supported by ANR-11-IDEX-0005-02 Laboratory of Excellence INFLAMEX and Fondation pour la Recherche Médicale (EQU201903007816).

## Conflict of Interest

AZ was employed by TRexBio.

The remaining authors declare that the research was conducted in the absence of any commercial or financial relationships that could be construed as a potential conflict of interest.
